# *catena*-Poly[[bis­(1-ethyl­imidazole-κ*N* ^3^)cobalt(II)]-μ-isophthalato-κ^2^ *O* ^1^:*O* ^3^]

**DOI:** 10.1107/S1600536808030778

**Published:** 2008-09-27

**Authors:** Juan Zhao

**Affiliations:** a College of Mechanical Engineering, Qingdao Technological University, Qingdao 266033, People’s Republic of China

## Abstract

In the title compound, [Co(C_8_H_4_O_4_)(C_5_H_8_N_2_)_2_]_*n*_, each cobalt(II) ion is coordinated by two N and two O atoms in a distorted tetra­hedral geometry. The isophthalate ligands bridge the metal ions to form polymeric zigzag chains extending along the *b* axis. Weak C—H⋯O inter­actions contribute to the crystal packing stability.

## Related literature

For the crystal structures of related copper and cobalt compounds, see: Song *et al.* (2007 ▶).
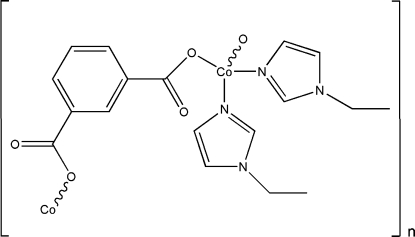


## Experimental

### 

#### Crystal data

[Co(C_8_H_4_O_4_)(C_5_H_8_N_2_)_2_]
*M* *_r_* = 415.31
Monoclinic, 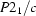

*a* = 15.174 (3) Å
*b* = 9.6650 (19) Å
*c* = 13.183 (3) Å
β = 104.63 (3)°
*V* = 1870.7 (7) Å^3^
*Z* = 4
Mo *K*α radiation
μ = 0.95 mm^−1^
*T* = 293 (2) K
0.20 × 0.10 × 0.10 mm

#### Data collection

Bruker SMART 1K CCD area-detector diffractometer
Absorption correction: multi-scan (*SADABS*; Sheldrick, 2004 ▶) *T*_min_ = 0.833, *T*_max_ = 0.911
3360 measured reflections
3276 independent reflections
2711 reflections with *I* > 2σ(*I*)
*R*_int_ = 0.034

#### Refinement

*R*[*F* ^2^ > 2σ(*F* ^2^)] = 0.057
*wR*(*F* ^2^) = 0.152
*S* = 1.02
3276 reflections
233 parameters
40 restraints
H-atom parameters constrained
Δρ_max_ = 0.73 e Å^−3^
Δρ_min_ = −1.49 e Å^−3^

### 

Data collection: *SMART* (Bruker, 2001 ▶); cell refinement: *SAINT* (Bruker, 2001 ▶); data reduction: *SAINT*; program(s) used to solve structure: *SHELXTL* (Sheldrick, 2008 ▶); program(s) used to refine structure: *SHELXTL*; molecular graphics: *SHELXTL*; software used to prepare material for publication: *SHELXTL*.

## Supplementary Material

Crystal structure: contains datablocks global, I. DOI: 10.1107/S1600536808030778/rz2249sup1.cif

Structure factors: contains datablocks I. DOI: 10.1107/S1600536808030778/rz2249Isup2.hkl

Additional supplementary materials:  crystallographic information; 3D view; checkCIF report

## Figures and Tables

**Table:** 

Co—O1	1.956 (3)
Co—N2	2.008 (4)
Co—O4^i^	2.008 (3)
Co—N4	2.035 (3)

**Table:** 

O1—Co—N2	118.23 (14)
O1—Co—O4^i^	115.92 (12)
N2—Co—O4^i^	104.31 (13)
O1—Co—N4	108.77 (13)
N2—Co—N4	109.39 (15)
O4^i^—Co—N4	98.31 (13)

Symmetry code: (i) 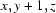.

**Table 2:** Hydrogen-bond geometry (Å, °)

*D*—H⋯*A*	*D*—H	H⋯*A*	*D*⋯*A*	*D*—H⋯*A*
C12—H12*A*⋯O1	0.93	2.45	2.768 (5)	100
C3—H3*A*⋯O3^ii^	0.93	2.40	3.260 (6)	154
C5—H5*A*⋯O3^iii^	0.93	2.41	3.327 (7)	168

Symmetry codes: (ii) 
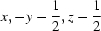
; (iii) 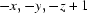.

**Fig. 1.**
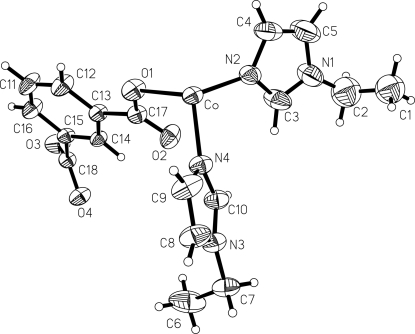
The asymmetric unit of the title compound showing the atom numbering scheme and 30% probability displacement ellipsoids.

**Fig. 2.**
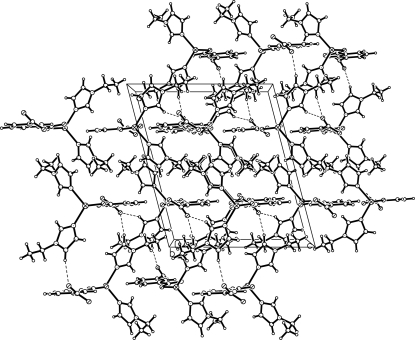
Packing diagram of the title compound viewed down the b axis.

## supplementary crystallographic information

### Comment

In the title compound (Fig. 1), the cobalt(II) ion is coordinated by two N and two O atoms in a distorted tetrahedral geometry. The values of bond distances and angles (Table 1) agree well with those observed in [Co(isophthalato)(1-*H*-imidazole)_2_] (Song, *et al.*, 2007). Each isophthalate dianion acts as a bidentate ligand to bridge two cobalt(II) atoms through the monodentate carboxylate groups, building a zigzag chain structure along the *b* axis (Fig. 2). The metal-metal distance across the polymer backbone is 9.665 (7) Å. Weak C—H···O hydrogen interactions contribute to the crystal packing stability (Table 2).

### Experimental

The reaction of CoCl_2_.6H_2_O (1.19 g, 5 mmol) with isophthalic acid (0.83 g, 5 mmol) in a water/ethanol (3:1 v/v) solution (40 ml) at 363 K for 30 minutes produced a blue solution, to which 1-ethylimidazole (0.84 g,10 mmol) was added. The reaction solution was kept at room temperature after stirring for an hour at 333 K. Pink crystals were obtained after a few days on slow evaporation of he solvent.

### Refinement

H atoms were positioned geometrically and allowed to ride on their parent atoms, with C—H = 0.93-0.97 Å and with *U*_iso_(H) = 1.2*U*_eq_(C) or 1.5*U*_eq_(C) for methyl H atoms.

### Crystal data

**Table:**

[Co(C_8_H_4_O_4_)(C_5_H_8_N_2_)_2_]	*F*(000) = 860
*M**_r_* = 415.31	*D*_x_ = 1.475 Mg m^−^^3^
Monoclinic, *P*2_1_/*c*	Mo *K*α radiation, λ = 0.71073 Å
Hall symbol: -P 2ybc	Cell parameters from 25 reflections
*a* = 15.174 (3) Å	θ = 10–14°
*b* = 9.6650 (19) Å	µ = 0.95 mm^−^^1^
*c* = 13.183 (3) Å	*T* = 293 K
β = 104.63 (3)°	Block, pink
*V* = 1870.7 (7) Å^3^	0.20 × 0.10 × 0.10 mm
*Z* = 4	

### Data collection

**Table:**

Bruker SMART 1K CCD area-detector diffractometer	3276 independent reflections
Radiation source: fine-focus sealed tube	2711 reflections with *I* > 2σ(*I*)
graphite	*R*_int_ = 0.034
Thin–slice ω scans	θ_max_ = 25.2°, θ_min_ = 1.4°
Absorption correction: multi-scan (SADABS; Sheldrick, 2004)	*h* = −18→17
*T*_min_ = 0.833, *T*_max_ = 0.911	*k* = 0→11
3360 measured reflections	*l* = 0→15

### Refinement

**Table:**

Refinement on *F*^2^	Secondary atom site location: difference Fourier map
Least-squares matrix: full	Hydrogen site location: inferred from neighbouring sites
*R*[*F*^2^ > 2σ(*F*^2^)] = 0.057	H-atom parameters constrained
*wR*(*F*^2^) = 0.152	*w* = 1/[σ^2^(*F*_o_^2^) + (0.08*P*)^2^ + 3.5*P*] where *P* = (*F*_o_^2^ + 2*F*_c_^2^)/3
*S* = 1.02	(Δ/σ)_max_ = 0.001
3276 reflections	Δρ_max_ = 0.73 e Å^−^^3^
233 parameters	Δρ_min_ = −1.49 e Å^−^^3^
40 restraints	Extinction correction: SHELXTL (Sheldrick, 2008), Fc^*^=kFc[1+0.001xFc^2^λ^3^/sin(2θ)]^-1/4^
Primary atom site location: structure-invariant direct methods	Extinction coefficient: 0.0109 (14)

### Special details

**Table:**

Geometry. All e.s.d.'s (except the e.s.d. in the dihedral angle between two l.s. planes) are estimated using the full covariance matrix. The cell e.s.d.'s are taken into account individually in the estimation of e.s.d.'s in distances, angles and torsion angles; correlations between e.s.d.'s in cell parameters are only used when they are defined by crystal symmetry. An approximate (isotropic) treatment of cell e.s.d.'s is used for estimating e.s.d.'s involving l.s. planes.
Refinement. Refinement of *F*^2^ against ALL reflections. The weighted *R*-factor *wR* and goodness of fit *S* are based on *F*^2^, conventional *R*-factors *R* are based on *F*, with *F* set to zero for negative *F*^2^. The threshold expression of *F*^2^ > σ(*F*^2^) is used only for calculating *R*-factors(gt) *etc*. and is not relevant to the choice of reflections for refinement. *R*-factors based on *F*^2^ are statistically about twice as large as those based on *F*, and *R*- factors based on ALL data will be even larger.

### Fractional atomic coordinates and isotropic or equivalent isotropic displacement parameters (Å^2^)

**Table:**

	*x*	*y*	*z*	*U*_iso_*/*U*_eq_	
Co	0.26136 (4)	0.30808 (5)	0.53367 (4)	0.0305 (2)	
O1	0.2699 (2)	0.1668 (3)	0.6419 (2)	0.0427 (8)	
N1	0.0386 (3)	0.3053 (5)	0.2750 (4)	0.0612 (12)	
C1	−0.0178 (5)	0.3839 (7)	0.0953 (5)	0.083	
H1A	−0.0469	0.3539	0.0254	0.124*	
H1B	0.0404	0.4237	0.0963	0.124*	
H1C	−0.0552	0.4518	0.1174	0.124*	
N2	0.1440 (2)	0.3249 (3)	0.4223 (3)	0.0393 (8)	
O2	0.2455 (2)	0.0313 (3)	0.5018 (2)	0.0454 (8)	
C2	−0.0054 (5)	0.2678 (8)	0.1649 (6)	0.089	
H2A	0.0318	0.1991	0.1415	0.107*	
H2B	−0.0642	0.2263	0.1617	0.107*	
N3	0.4528 (2)	0.2000 (4)	0.3686 (3)	0.0393 (8)	
C3	0.1228 (3)	0.2707 (5)	0.3287 (4)	0.0561 (13)	
H3A	0.1616	0.2145	0.3024	0.067*	
O3	0.1949 (2)	−0.5482 (3)	0.6821 (3)	0.0489 (8)	
N4	0.3661 (2)	0.2814 (3)	0.4648 (3)	0.0351 (8)	
C4	0.0695 (4)	0.4016 (6)	0.4269 (5)	0.0667 (15)	
H4A	0.0651	0.4550	0.4841	0.080*	
O4	0.2898 (2)	−0.4975 (3)	0.5853 (2)	0.0401 (7)	
C5	0.0032 (4)	0.3894 (7)	0.3373 (5)	0.0772 (18)	
H5A	−0.0542	0.4298	0.3216	0.093*	
C6	0.5078 (4)	−0.0300 (6)	0.3365 (6)	0.0754 (18)	
H6A	0.5275	−0.0854	0.2859	0.113*	
H6B	0.4559	−0.0727	0.3529	0.113*	
H6C	0.5563	−0.0226	0.3991	0.113*	
C7	0.4823 (3)	0.1117 (5)	0.2922 (4)	0.0512 (12)	
H7A	0.5342	0.1537	0.2738	0.061*	
H7B	0.4333	0.1042	0.2289	0.061*	
C8	0.5036 (3)	0.2923 (5)	0.4353 (4)	0.0535 (13)	
H8A	0.5639	0.3162	0.4400	0.064*	
C9	0.4501 (3)	0.3430 (5)	0.4934 (4)	0.0506 (12)	
H9A	0.4674	0.4100	0.5452	0.061*	
C10	0.3710 (3)	0.1963 (4)	0.3889 (3)	0.0386 (10)	
H10A	0.3232	0.1402	0.3536	0.046*	
C11	0.2681 (4)	−0.1647 (4)	0.8394 (3)	0.0476 (12)	
H11A	0.2741	−0.1501	0.9105	0.057*	
C12	0.2682 (3)	−0.0546 (4)	0.7746 (3)	0.0434 (11)	
H12A	0.2733	0.0347	0.8018	0.052*	
C13	0.2608 (3)	−0.0744 (4)	0.6689 (3)	0.0303 (8)	
C14	0.2555 (3)	−0.2084 (4)	0.6290 (3)	0.0283 (8)	
H14A	0.2528	−0.2226	0.5585	0.034*	
C15	0.2541 (3)	−0.3208 (4)	0.6941 (3)	0.0284 (8)	
C16	0.2593 (3)	−0.2984 (4)	0.7997 (3)	0.0423 (11)	
H16A	0.2568	−0.3728	0.8436	0.051*	
C17	0.2589 (3)	0.0479 (4)	0.5977 (3)	0.0318 (9)	
C18	0.2449 (3)	−0.4664 (4)	0.6524 (3)	0.0309 (8)	

### Atomic displacement parameters (Å^2^)

**Table:**

	*U*^11^	*U*^22^	*U*^33^	*U*^12^	*U*^13^	*U*^23^
Co	0.0457 (4)	0.0143 (3)	0.0364 (3)	0.0020 (2)	0.0193 (2)	0.0006 (2)
O1	0.072 (2)	0.0137 (13)	0.0492 (17)	0.0018 (13)	0.0275 (16)	0.0019 (12)
N1	0.053 (2)	0.061 (3)	0.063 (3)	0.017 (2)	0.0013 (19)	−0.015 (2)
C1	0.083	0.083	0.083	0.000	0.024	0.000
N2	0.047 (2)	0.0293 (18)	0.0450 (19)	0.0059 (15)	0.0176 (16)	0.0005 (15)
O2	0.071 (2)	0.0290 (15)	0.0397 (16)	−0.0045 (14)	0.0194 (14)	0.0074 (13)
C2	0.089	0.089	0.089	0.000	0.022	0.000
N3	0.045 (2)	0.0357 (19)	0.0419 (19)	−0.0005 (16)	0.0201 (16)	−0.0050 (16)
C3	0.047 (3)	0.052 (3)	0.066 (3)	0.012 (2)	0.009 (2)	−0.021 (2)
O3	0.066 (2)	0.0172 (14)	0.075 (2)	−0.0053 (14)	0.0375 (18)	0.0017 (14)
N4	0.045 (2)	0.0225 (16)	0.0430 (19)	0.0001 (14)	0.0211 (16)	−0.0021 (14)
C4	0.061 (3)	0.079 (4)	0.064 (3)	0.027 (3)	0.023 (2)	−0.013 (3)
O4	0.069 (2)	0.0155 (13)	0.0435 (16)	0.0009 (13)	0.0284 (15)	−0.0034 (12)
C5	0.058 (3)	0.087 (4)	0.084 (4)	0.028 (3)	0.015 (3)	−0.012 (3)
C6	0.074 (4)	0.046 (3)	0.120 (5)	0.007 (3)	0.050 (4)	−0.017 (3)
C7	0.053 (3)	0.053 (3)	0.055 (3)	0.006 (2)	0.027 (2)	−0.013 (2)
C8	0.047 (3)	0.061 (3)	0.060 (3)	−0.017 (2)	0.028 (2)	−0.014 (3)
C9	0.063 (3)	0.043 (3)	0.054 (3)	−0.022 (2)	0.031 (2)	−0.017 (2)
C10	0.043 (2)	0.030 (2)	0.047 (2)	−0.0020 (18)	0.0191 (19)	−0.0082 (19)
C11	0.088 (4)	0.030 (2)	0.029 (2)	0.003 (2)	0.024 (2)	−0.0025 (18)
C12	0.082 (3)	0.0154 (18)	0.038 (2)	0.001 (2)	0.025 (2)	−0.0050 (17)
C13	0.045 (2)	0.0152 (17)	0.033 (2)	0.0021 (16)	0.0156 (17)	0.0009 (15)
C14	0.041 (2)	0.0208 (18)	0.0267 (18)	0.0020 (16)	0.0141 (16)	−0.0008 (15)
C15	0.039 (2)	0.0169 (18)	0.033 (2)	0.0019 (15)	0.0156 (16)	−0.0003 (15)
C16	0.076 (3)	0.022 (2)	0.036 (2)	0.003 (2)	0.026 (2)	0.0046 (17)
C17	0.043 (2)	0.0185 (18)	0.038 (2)	−0.0002 (16)	0.0164 (17)	0.0023 (16)
C18	0.045 (2)	0.0134 (17)	0.037 (2)	0.0051 (16)	0.0143 (17)	0.0026 (15)

### Geometric parameters (Å, °)

**Table:**

Co—O1	1.956 (3)	O4—C18	1.281 (5)
Co—N2	2.008 (4)	O4—Co^ii^	2.008 (3)
Co—O4^i^	2.008 (3)	C5—H5A	0.9300
Co—N4	2.035 (3)	C6—C7	1.501 (8)
O1—C17	1.281 (5)	C6—H6A	0.9600
N1—C3	1.337 (7)	C6—H6B	0.9600
N1—C5	1.358 (7)	C6—H6C	0.9600
N1—C2	1.481 (8)	C7—H7A	0.9700
C1—C2	1.432 (9)	C7—H7B	0.9700
C1—H1A	0.9600	C8—C9	1.341 (7)
C1—H1B	0.9600	C8—H8A	0.9300
C1—H1C	0.9600	C9—H9A	0.9300
N2—C3	1.303 (6)	C10—H10A	0.9300
N2—C4	1.366 (6)	C11—C12	1.365 (6)
O2—C17	1.239 (5)	C11—C16	1.387 (6)
C2—H2A	0.9700	C11—H11A	0.9300
C2—H2B	0.9700	C12—C13	1.382 (6)
N3—C10	1.336 (6)	C12—H12A	0.9300
N3—C8	1.349 (6)	C13—C14	1.393 (5)
N3—C7	1.473 (6)	C13—C17	1.505 (5)
C3—H3A	0.9300	C14—C15	1.387 (5)
O3—C18	1.227 (5)	C14—H14A	0.9300
N4—C10	1.312 (5)	C15—C16	1.391 (6)
N4—C9	1.370 (6)	C15—C18	1.505 (5)
C4—C5	1.350 (8)	C16—H16A	0.9300
C4—H4A	0.9300		
			
O1—Co—N2	118.23 (14)	H6A—C6—H6B	109.5
O1—Co—O4^i^	115.92 (12)	C7—C6—H6C	109.5
N2—Co—O4^i^	104.31 (13)	H6A—C6—H6C	109.5
O1—Co—N4	108.77 (13)	H6B—C6—H6C	109.5
N2—Co—N4	109.39 (15)	N3—C7—C6	110.8 (4)
O4^i^—Co—N4	98.31 (13)	N3—C7—H7A	109.5
C17—O1—Co	108.5 (2)	C6—C7—H7A	109.5
C3—N1—C5	107.5 (5)	N3—C7—H7B	109.5
C3—N1—C2	126.2 (5)	C6—C7—H7B	109.5
C5—N1—C2	126.2 (5)	H7A—C7—H7B	108.1
C2—C1—H1A	109.5	C9—C8—N3	106.6 (4)
C2—C1—H1B	109.5	C9—C8—H8A	126.7
H1A—C1—H1B	109.5	N3—C8—H8A	126.7
C2—C1—H1C	109.5	C8—C9—N4	109.9 (4)
H1A—C1—H1C	109.5	C8—C9—H9A	125.1
H1B—C1—H1C	109.5	N4—C9—H9A	125.1
C3—N2—C4	104.5 (4)	N4—C10—N3	111.8 (4)
C3—N2—Co	128.4 (3)	N4—C10—H10A	124.1
C4—N2—Co	127.0 (3)	N3—C10—H10A	124.1
C1—C2—N1	113.0 (6)	C12—C11—C16	120.4 (4)
C1—C2—H2A	109.0	C12—C11—H11A	119.8
N1—C2—H2A	109.0	C16—C11—H11A	119.8
C1—C2—H2B	109.0	C11—C12—C13	120.6 (4)
N1—C2—H2B	109.0	C11—C12—H12A	119.7
H2A—C2—H2B	107.8	C13—C12—H12A	119.7
C10—N3—C8	107.1 (4)	C12—C13—C14	119.5 (3)
C10—N3—C7	125.4 (4)	C12—C13—C17	120.3 (3)
C8—N3—C7	127.4 (4)	C14—C13—C17	120.3 (3)
N2—C3—N1	112.1 (4)	C15—C14—C13	120.2 (3)
N2—C3—H3A	124.0	C15—C14—H14A	119.9
N1—C3—H3A	124.0	C13—C14—H14A	119.9
C10—N4—C9	104.7 (3)	C14—C15—C16	119.4 (3)
C10—N4—Co	128.3 (3)	C14—C15—C18	121.4 (3)
C9—N4—Co	126.8 (3)	C16—C15—C18	119.2 (3)
C5—C4—N2	110.8 (5)	C11—C16—C15	119.8 (4)
C5—C4—H4A	124.6	C11—C16—H16A	120.1
N2—C4—H4A	124.6	C15—C16—H16A	120.1
C18—O4—Co^ii^	110.3 (2)	O2—C17—O1	123.3 (3)
C4—C5—N1	105.1 (5)	O2—C17—C13	120.5 (3)
C4—C5—H5A	127.4	O1—C17—C13	116.2 (3)
N1—C5—H5A	127.4	O3—C18—O4	123.3 (4)
C7—C6—H6A	109.5	O3—C18—C15	119.6 (3)
C7—C6—H6B	109.5	O4—C18—C15	117.1 (3)
			
N2—Co—O1—C17	61.4 (3)	N3—C8—C9—N4	1.0 (6)
O4^i^—Co—O1—C17	−173.7 (3)	C10—N4—C9—C8	−0.8 (6)
N4—Co—O1—C17	−64.1 (3)	Co—N4—C9—C8	175.2 (3)
O1—Co—N2—C3	−95.9 (4)	C9—N4—C10—N3	0.4 (5)
O4^i^—Co—N2—C3	133.6 (4)	Co—N4—C10—N3	−175.6 (3)
N4—Co—N2—C3	29.3 (5)	C8—N3—C10—N4	0.2 (5)
O1—Co—N2—C4	87.1 (4)	C7—N3—C10—N4	176.4 (4)
O4^i^—Co—N2—C4	−43.4 (5)	C16—C11—C12—C13	−1.0 (8)
N4—Co—N2—C4	−147.7 (4)	C11—C12—C13—C14	−1.6 (7)
C3—N1—C2—C1	−110.4 (7)	C11—C12—C13—C17	178.7 (4)
C5—N1—C2—C1	64.5 (9)	C12—C13—C14—C15	2.4 (6)
C4—N2—C3—N1	−1.7 (6)	C17—C13—C14—C15	−177.8 (4)
Co—N2—C3—N1	−179.2 (3)	C13—C14—C15—C16	−0.8 (6)
C5—N1—C3—N2	1.0 (7)	C13—C14—C15—C18	177.7 (4)
C2—N1—C3—N2	176.6 (5)	C12—C11—C16—C15	2.6 (8)
O1—Co—N4—C10	83.3 (4)	C14—C15—C16—C11	−1.7 (7)
N2—Co—N4—C10	−47.2 (4)	C18—C15—C16—C11	179.8 (4)
O4^i^—Co—N4—C10	−155.7 (4)	Co—O1—C17—O2	0.2 (5)
O1—Co—N4—C9	−91.9 (4)	Co—O1—C17—C13	−178.5 (3)
N2—Co—N4—C9	137.7 (4)	C12—C13—C17—O2	−174.6 (4)
O4^i^—Co—N4—C9	29.2 (4)	C14—C13—C17—O2	5.6 (6)
C3—N2—C4—C5	1.8 (7)	C12—C13—C17—O1	4.0 (6)
Co—N2—C4—C5	179.4 (4)	C14—C13—C17—O1	−175.7 (4)
N2—C4—C5—N1	−1.3 (8)	Co^ii^—O4—C18—O3	2.6 (5)
C3—N1—C5—C4	0.2 (7)	Co^ii^—O4—C18—C15	−176.4 (3)
C2—N1—C5—C4	−175.5 (6)	C14—C15—C18—O3	−138.0 (4)
C10—N3—C7—C6	−78.9 (6)	C16—C15—C18—O3	40.5 (6)
C8—N3—C7—C6	96.6 (6)	C14—C15—C18—O4	41.0 (6)
C10—N3—C8—C9	−0.7 (6)	C16—C15—C18—O4	−140.5 (4)
C7—N3—C8—C9	−176.8 (5)		

Symmetry codes: (i) *x*, *y*+1, *z*; (ii) *x*, *y*−1, *z*.

### Hydrogen-bond geometry (Å, °)

**Table:**

*D*—H···*A*	*D*—H	H···*A*	*D*···*A*	*D*—H···*A*
C12—H12A···O1	0.93	2.45	2.768 (5)	100
C3—H3A···O3^iii^	0.93	2.40	3.260 (6)	154
C5—H5A···O3^iv^	0.93	2.41	3.327 (7)	168

Symmetry codes: (iii) *x*, −*y*−1/2, *z*−1/2; (iv) −*x*, −*y*, −*z*+1.
